# High-throughput genotyping of CRISPR/Cas9-mediated mutants using fluorescent PCR-capillary gel electrophoresis

**DOI:** 10.1038/srep15587

**Published:** 2015-10-26

**Authors:** Muhammad Khairul Ramlee, Tingdong Yan, Alice M. S. Cheung, Charles T. H. Chuah, Shang Li

**Affiliations:** 1 Cancer and Stem Cell Biology Program, Duke-NUS Graduate Medical School, Singapore 169857; 2 Department of Haematology, Singapore General Hospital, Singapore 169608; 3 Department of Physiology, Yong Loo Lin School of Medicine, National University of Singapore, Singapore 117597.

## Abstract

Recent advances in the engineering of sequence-specific synthetic nucleases provide enormous opportunities for genetic manipulation of gene expression in order to study their cellular function *in vivo*. However, current genotyping methods to detect these programmable nuclease-induced insertion/deletion (indel) mutations in targeted human cells are not compatible for high-throughput screening of knockout clones due to inherent limitations and high cost. Here, we describe an efficient method of genotyping clonal CRISPR/Cas9-mediated mutants in a high-throughput manner involving the use of a direct lysis buffer to extract crude genomic DNA straight from cells in culture, and fluorescent PCR coupled with capillary gel electrophoresis. This technique also allows for genotyping of multiplexed gene targeting in a single clone. Overall, this time- and cost-saving technique is able to circumvent the limitations of current genotyping methods and support high-throughput screening of nuclease-induced mutants.

Genome editing is an invaluable technique in modern day genetics. Targeted modification of mammalian genome has greatly improved in the past decade or so particularly with the introduction of programmable nucleases such as transcription activator-like effector nucleases (TALENs), zinc-finger nucleases (ZFNs) and the more recently described RNA-guided clustered regularly interspaced short palindromic repeats (CRISPR)/Cas9 system. An integral function of these programmable nucleases is the targeted knockout of specific genes via the introduction of insertion/deletion (indel) mutations in the coding region of these genes. These potentially frameshift-causing mutations are mediated by the error-prone non-homologous end joining (NHEJ) repair induced by double strand breaks (DSBs) by the nucleases. The resultant frameshift may, in turn, lead to loss of gene expression due to premature termination of translation and nonsense-mediated decay^1^,^2^,^3^. Due to its ease of use and low cost, the CRISPR/Cas9 system has made it extremely simple to specifically knock out virtually any genes in the human genome.

Despite the convenience of design offered by the CRISPR/Cas9 system, genotyping of mutated clones remains largely a bottleneck, especially for high-throughput purposes^4^,^5^. There are currently several techniques available for detecting engineered nuclease-mediated mutations in cells: SURVEYOR or T7E1 assay which detects mismatches in double-stranded DNA^6^,^7^; DNA melting analysis which differentiates fragments based on their melting curve^8^,^9^; restriction fragment length polymorphism (RFLP) assay which reports the disappearance of restriction sequences at nuclease target sites^10^; and Sanger and deep sequencing. However, each of these techniques suffers certain drawbacks that hamper their use in a high-throughput format.

SURVEYOR and T7E1 assays, whilst cheap and easy to use, are unable to differentiate between identically mutated alleles (homozygous mutants)^7^, thus mutants bearing these alleles are erroneously reported as wildtype clones. The simplicity and cost effectiveness of the RFLP assay are often outweighed by its lack of appropriate restriction sites^11^. DNA melting analysis, on one hand, is a relatively simple method to use but on the other, tends to be inconsistent and is not very informative. Lastly, sequencing methods, which can be said to be the gold standard for genotyping mutants, is extremely informative but rather costly especially for high-throughput purposes.

Here, we describe a technique that is able to circumvent the abovementioned limitations and is thus amenable to high-throughput detection of nuclease-induced mutants. Using fluorescent PCR coupled with capillary gel electrophoresis, we are able to effectively and efficiently screen for CRISPR/Cas9-mediated mutant clones harbouring indels at three targeted genes (*ATRX*, *TP53* and *MIR615*) in a high-throughput fashion. This technique also supports multiplexed targeting of genes which is particularly useful in synergistic and redundancy studies. Overall, the method we describe here is not merely an alternative to the existing genotyping techniques available but a superior one on the basis that it allows for high-throughput screening of mutants, which renders it more time- and cost-effective.

## Results

### Testing sgRNA targeting efficiency

**Figure 1:**
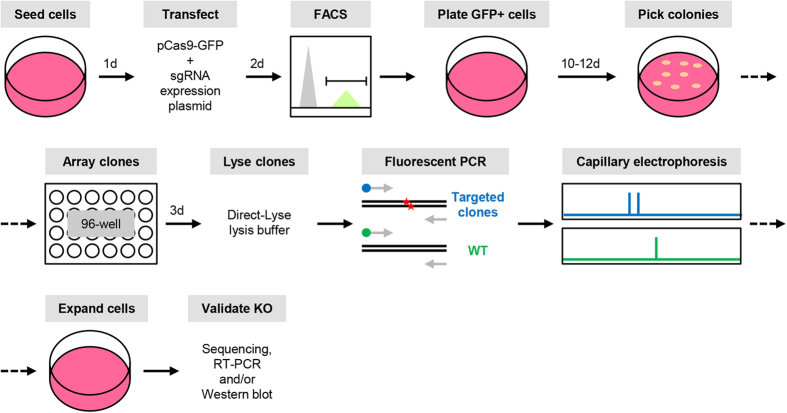
Schematic of high-throughput genotyping technique via fluorescent PCR-capillary gel electrophoresis. First, cells are transfected with plasmids expressing Cas9-GFP fusion protein and individual sgRNA. Two days later, GFP-positive cells are sorted and plated onto 10-cm dishes. When individual colonies of cells are visible, they are picked and arrayed on 96-well plates. When the arrayed cells reach ~80% confluence, they are lysed directly using Direct-Lyse buffer and the crude lysate is used to amplify the genomic region containing the expected indel site using fluorophore-labelled primers. The labelled amplicons are resolved via capillary gel electrophoresis and successful mutants are identified by shifts in fragment size with respect to wildtype fragment. Putative knock-out clones are expanded and validated via Sanger sequencing, quantitative RT-PCR and/or Western blot analysis.

Current methods of genotyping engineered nuclease-mediated mutations are not optimal for high-throughput screening of individual knockout clones due to inherent limitations and/or high cost. We thus sought to determine whether fluorescent PCR coupled with capillary gel electrophoresis is able to accurately genotype mutant clones in a high-throughput and cost-effective manner and hence circumvent the abovementioned limitations (see Fig. 1 for schematic of protocol).

We selected the near-diploid colorectal carcinoma cells, HCT116, as our model system, and chose three different genes found on three disparate chromosomes to test our hypothesis. *ATRX* encodes for a chromatin remodelling protein and is found on the X-chromosome^12^,^13^. *TP53* gene encodes the protein p53 which is crucial in regulating DNA repair, cell cycle progression and apoptosis^14^,^15^,^16^. This gene is found on chromosome 17 in humans^17^. Lastly, the *MIR615* gene encodes two microRNAs—miR615-5p and miR615-3p—and has been implicated in prostate and colon cancer^18^. This non-coding RNA gene is found on chromosome 12 in humans. For our gene targeting experiments, we focused mainly on the inactivation of miR615-3p, but has also investigated the effect of the genome targeting on miR615-5p expression.

**Figure 2:**
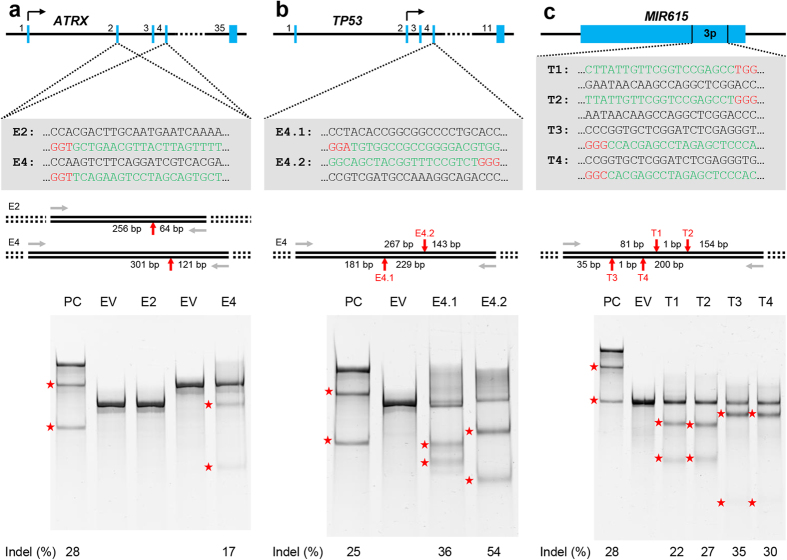
SURVEYOR mutation detection assay to test for sgRNA targeting efficiency. Specific regions of *ATRX* (**a**), *TP53* (**b**) and *MIR615* (**c**) genes were targeted using CRISPR/Cas9 system (top) and the efficiency of the sgRNA used were examined via SURVEYOR assay (bottom). Top: The exons/coding region of each gene are represented by blue boxes and are numbered accordingly. CRISPR/Cas9 target sequences are given in green (protospacer) and red (protospacer adjacent motif, PAM) and the corresponding name of the sgRNA are shown in bold. Middle: Grey arrows represent PCR primers used to amplify targeted regions. Red arrows indicate the expected cleavage site of each sgRNA target and the expected sizes of the cleavage product are given next to them. Bottom: SURVEYOR mutation detection assay results for each sgRNA tested. Red stars indicate the cleavage products of the samples indicated above each lane and the numbers at the bottom indicate estimated indel frequency. PC: positive control (G and C control from SURVEYOR assay kit); EV: empty vector (without sgRNA expression cassette).

We used the database set up by Feng Zhang and colleagues (http://tools.genome-engineering.org)^19^ to search for CRISPR single guide RNA (sgRNA) targets in exon 2 and 4 of *ATRX* gene and the region encoding miR615-3p in *MIR615* gene and ultimately chose two and four targets, respectively, which we named sgATRX-E2, sgATRX-E4 and sgMIR615-3p-T1, -T2, -T3 and -T4. We obtained two sgRNA plasmids targeting exon 4 of *TP53* gene from Voorhoeve’s laboratory and these were named sgTP53-E4.1 and –E4.2. In order to ascertain their efficiency in generating site-specific double-strand breaks, we transiently transfected HEK293 or HCT116 cells with pCas9WT-2A-GFP plasmid (Addgene) together with one of the sgRNA expression plasmid, and performed SURVEYOR mutation detection assay on purified genomic DNA derived from the transfected cells. Of the two *ATRX* sgRNA, sgATRX-E4 displayed efficient targeted cleavage whereas sgATRX-E2 did not show any activity within the detection limit of the SURVEYOR mutation detection assay (Fig. 2a). In contrast, all sgRNAs targeting *TP53* and *MIR615-3p* displayed high cutting efficiency (Fig. 2b,c). Hence, virtually all the sgRNAs tested were efficacious in mediating the cleavage of DNA at specific targets in the genome.

### Testing direct lysis condition

An important factor that renders a method compatible for high-throughput genotyping of CRISPR/Cas9-induced mutant cells is its capacity for easy and fast extraction of genomic DNA from individual clones. Thus, we were interested to test whether we could perform direct cell lysis to obtain crude genomic DNA and utilize it to amplify specific regions via PCR. We adapted a protocol originally reported for use with plant cells^20^ and tested its efficacy in the extraction of intact genomic DNA directly from human cells in culture. We concocted the Direct-Lyse buffer and performed preliminary tests to compare the optimum dilution factor for use with cultured human cells. Lysis of cells involves physical agitation of trypsinised cell suspension via trituration and subjecting the lysate to thermal cycling (see Materials and Methods for details). As compared to the original report involving plant cells which used 2× dilution factor, we discovered that 0.5× Direct-Lyse buffer (10 mM Tris pH 8.0, 2.5 mM EDTA, 0.2 M NaCl, 0.15% SDS, 0.3% Tween-20) worked optimally for the H1 human embryonic stem cells tested (Supplementary Figure S2a). We also found that the Direct-Lyse buffer is superior to sodium hydroxide lysis buffer (50 mM NaOH, 0.4 mM EDTA) which has been previously used for the same purpose^21^ (Supplementary Figure S2b). Moreover, lysates are stable for months if stored at lower than −20 °C, without any noticeable loss of quality as PCR template (data not shown). Lastly, in order to ascertain its compatibility, we performed PCR analysis of genomic regions spanning our genes of interest and found that they were amplified efficiently and consistently (Supplementary Figure S2c). Hence with the use of our homemade lysis buffer, Direct-Lyse, we were able to obviate the need to expand clones before extraction of genomic DNA, saving both time and cost in the process.

### Genotyping clones with fluorescent capillary gel electrophoresis

**Figure 3:**
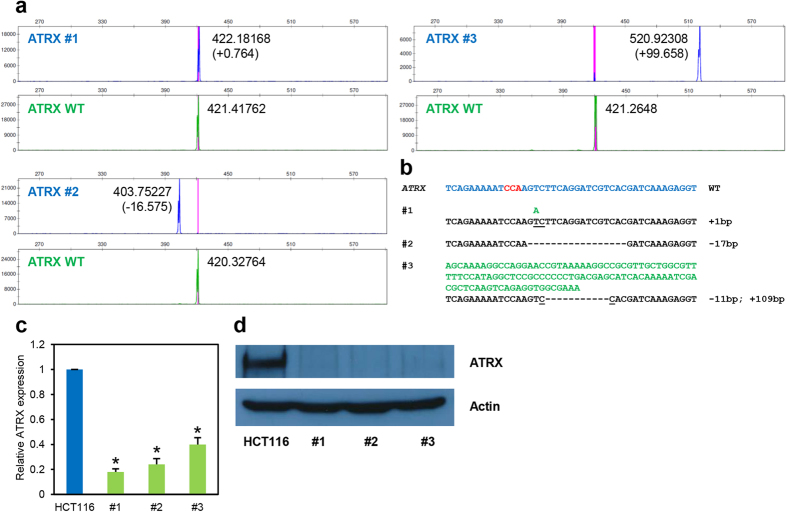
Genotyping of *ATRX*-targeted clones via fluorescent PCR-capillary gel electrophoresis. (**a**) HCT116 cells targeted with sgATRX-E4 were genotyped using fluorescent PCR-capillary gel electrophoresis and three representative clones are shown. Blue peaks indicate fragments obtained from PCR amplification of the region spanning the sgRNA target site in targeted cells using 6-FAM-labeled primers. Green peaks indicate similar fragments but from wildtype parental HCT116 cells and thus act as an internal size control. The numbers given in each plot represent the sizes of each peak (or fragment) and those in parentheses are the calculated difference in size (in base pairs) with respect to individual wildtype peaks. (**b**) Sanger sequencing results for the individual clones. Wildtype sequence is shown in blue and the PAM sequence in red. Inserted nucleotides are shown in green and underlined are the flanking nucleotides in the original sequence. Deleted nucleotides are shown as dashes (−). Quantitative RT-PCR (**c**) and Western blot (**d**) analyses were performed to corroborate knockout status of the clones shown. Error bars represent standard deviations of values from two independent experiments (n = 2). Asterisks represent significantly different (p < 0.05) expression levels as compared to the wildtype parental clone using one-tailed t-test.

**Figure 4:**
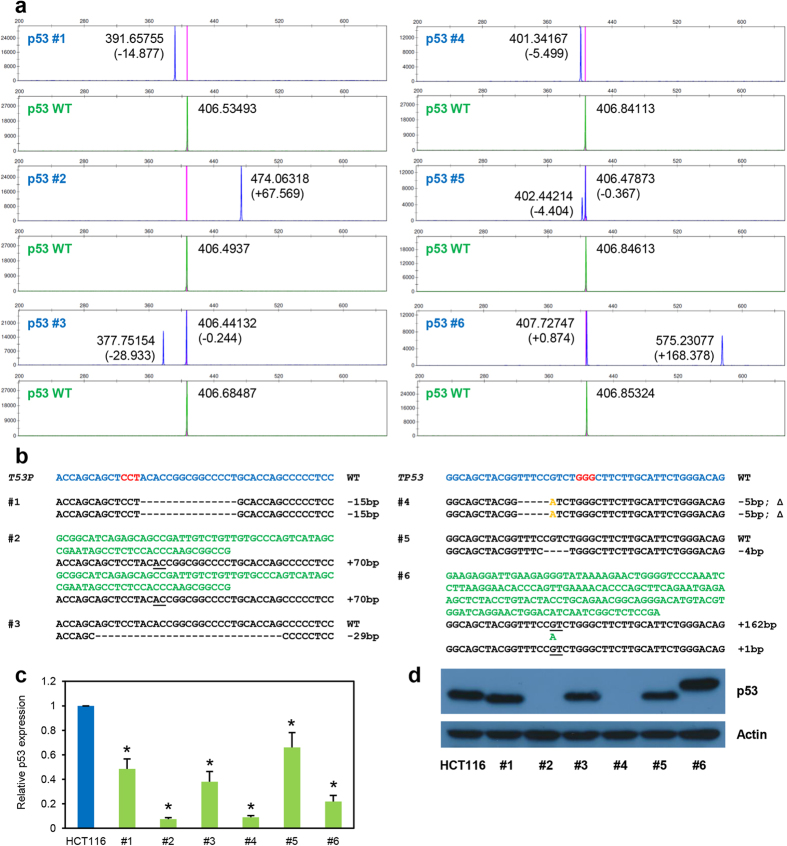
Genotyping of *TP53*-targeted clones via fluorescent PCR-capillary gel electrophoresis. *TP53*-targeted HCT116 cells were genotyped using fluorescent PCR-capillary gel electrophoresis (**a**) and several representative clones are shown. Clones #1 to #3 and clones #4 to #6 were targeted by sgTP53-E4.1 and –E4.2, respectively. (**b**) Sanger sequencing was performed to validate the indel mutations harboured by each clones. Representation of mutations is similar to that in Fig. 3b; in addition, orange nucleotides indicate substituted bases. Quantitative RT-PCR (**c**) and Western blot (**d**) analyses were performed to confirm the genotype of the clones. Error bars represent standard deviations of values from two independent experiments (n = 2). Asterisks represent significantly different (p < 0.05) expression levels as compared to the wildtype parental clone using one-tailed t-test.

**Figure 5:**
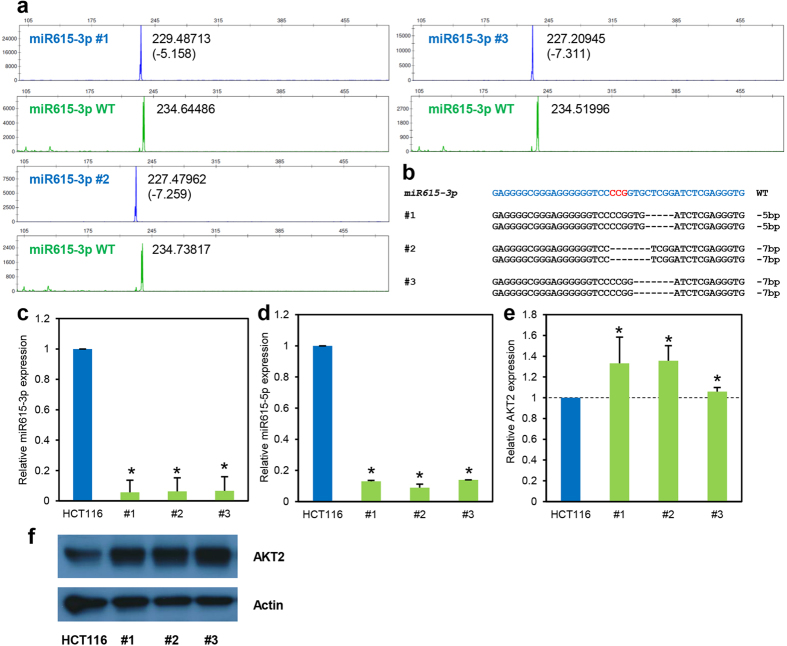
Genotyping of MIR615-3p-targeted clones via fluorescent PCR-capillary gel electrophoresis. *MIR615* gene (specifically the region encoding miR615-3p) was targeted using CRISPR/Cas9 system in HCT116 cells. The genotype of individual targeted clones was determined via fluorescent PCR coupled with capillary gel electrophoresis (**a**) and verified using Sanger sequencing (**b**) and quantitative RT-PCR (**c**). (**d**) The expression of miR615-5p was evaluated using quantitative RT-PCR. In addition, the expression of a known target of miR615-3p, *AKT2*, was examined using quantitative RT-PCR (**e**) and Western blot analysis (**f**). All symbols and representations are identical to those in Fig. 3. Error bars represent standard deviations of values from two independent experiments (n = 2). Asterisks represent significantly different (p < 0.05) expression levels as compared to the wildtype parental clone using one-tailed t-test.

**Figure 6:**
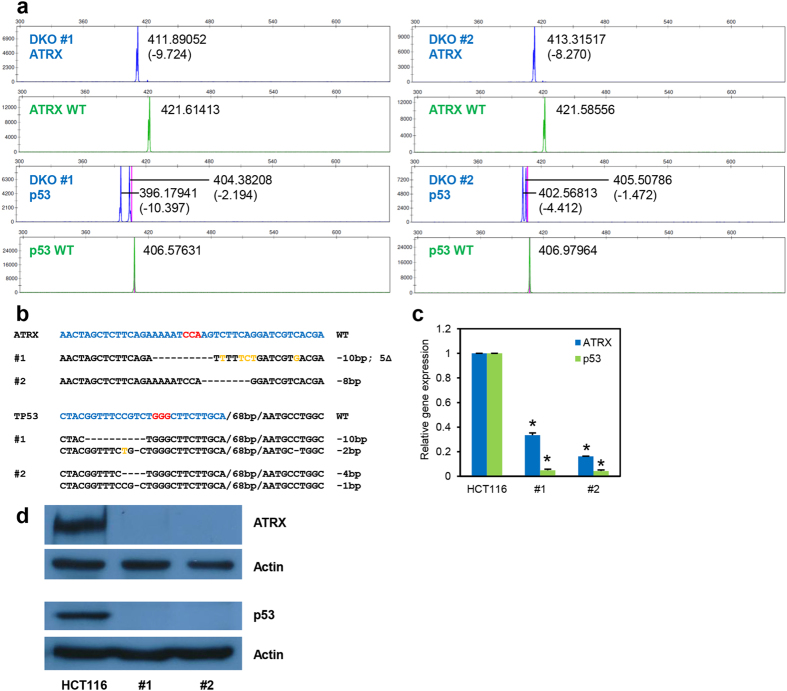
Genotyping of multiplex targeted clones via fluorescent PCR-capillary gel electrophoresis. The genome of HCT116 cells was targeted using sgATRX-E4 and sgTP53-E4.2 and clones were genotyped via fluorescent PCR-capillary gel electrophoresis (**a**) at both loci (exon 4 of *ATRX* gene and exon 4 of *TP53* gene). Two double knockout clones are shown. Sanger sequencing (**b**), quantitative RT-PCR (**c**) and Western blot (**d**) analyses were performed to validate the fluorescent PCR-capillary gel electrophoresis results and double knockout status of the two clones. All symbols and representations are identical to those in Figs 3 and 4. Error bars represent standard deviations of values from two independent experiments (n = 2). Asterisks represent significantly different (p < 0.05) expression levels as compared to the wildtype parental clone using one-tailed t-test.

Fluorescent capillary gel electrophoresis has previously been used with CRISPR/Cas9-mediated genome disruption to measure its cutting efficiency^22^. Taking into consideration its high sensitivity, we sought to assess this technique for its ability to accurately and efficiently genotype CRISPR/Cas9-induced insertion/deletion (indel) mutations in the targeted cells. We examined *ATRX*, *TP53* and *MIR615* in near-diploid HCT116 colorectal carcinoma cells for this purpose. We first cultured HCT116 cells and co-transfected them with pCas9WT-2A-GFP plasmid and individual sgRNA expression plasmids targeting *ATRX* exon 4 (sgATRX-E4), *TP53* exon 4 (sgTP53E4.1 and sgTP53E4.2) and *MIR615* region encoding miR615-3p (sgMIR615-3p-T1 to -T4). Two days after the transient transfection, we performed FACS to sort for GFP-positive cells and plated them on 10-cm dish at 500–2,500 cells per dish. When individual colonies were large enough (about 2–3 mm in diameter or 500–1,000 cells per colony), we picked and arrayed them on 96-well plates. When these clones reached about 80% confluence, we lysed them with 0.5× Direct-Lyse buffer and used the lysate directly to amplify genomic regions spanning the sgRNA target sites with 6-FAM-labelled PCR primers. Lysates of untransfected parental cells were subjected to amplification with HEX-labelled oligonucleotides in parallel as a control. The 6-FAM- and HEX-labelled amplicons were diluted accordingly and mixed in equal parts before being resolved via capillary gel electrophoresis (CGE) on Applied Biosystems 3500xL Genetic Analyzer. With HEX-labelled wildtype DNA fragment as reference (green channel in CGE program), targeted clones with indel mutations are expected to show shifted peaks in the blue channel corresponding to the 6-FAM-labelled fragments due to their altered fragment size (see Figs 1 and 3, 4, 5, 6).

Since *ATRX* gene is found on the X chromosome and HCT116 cell line is derived from a male patient^23^, its mutants are expected to show a single offset peak. As expected, using sgATRX-E4, 17 out of 26 clones (65.4%) showed a shift in fragment size ranging from deletion or insertion of one to more than 90 base pairs (Fig. 3a; Supplementary Figure S3a; Supplementary Table 1; and data not shown). We sequenced several representative clones and found that indels reported by the fluorescent capillary electrophoresis method tally robustly with the sequencing results (Fig. 3b; and Supplementary Figure S3b). In addition, these clones displayed strong attenuation of mRNA expression (Fig. 3c) likely due to nonsense-mediated decay, and complete ablation of protein expression as shown by western blot analysis (Fig. 3d). Of note, clone #1 had one nucleotide insertion (Fig. 3a,b) and this small change in size was readily and accurately detected by the fluorescent capillary electrophoresis technique.

Next, we applied the fluorescent capillary electrophoresis technique to examine the CRISPR/Cas9-mediated targeting efficiency of a bi-allelic gene in HCT116 cells—*TP53*. We anticipated the technique to rigorously discern between wildtype clones, heterozygous mutants (clones containing a wildtype and a mutant allele), compound heterozygous mutants (clones with two mutant alleles which are non-identical) and homozygous mutants (clones comprising of two identical mutant alleles). As expected, targeting with sgTP53-E4.1 and sgTP53-E4.2 produced 4.7% and 5.3% heterozygous mutants, 34.9% and 34.2% compound heterozygous mutants, and 39.5% and 44.7% homozygous mutants, respectively (Supplementary Table 1). These mutant clones harboured indels ranging from one to more than 200 base pairs (Fig. 4a; Supplementary Figure S3c; and data not shown). We picked a few clones for each sgRNA and sequenced their genome to ascertain the accuracy of the fluorescent capillary electrophoresis results (Fig. 4b; and Supplementary Figure S3d). As with *ATRX* gene targeting, the fluorescent capillary electrophoresis method is very accurate in predicting shorter indels (<30 bp) but tend to overestimate larger indels (>30 bp) (Fig. 4a,b; Supplementary Figures S3c,3d; and data not shown). Nevertheless, qRT-PCR and western blot analyses corroborate the authenticity of the genotype of these clones as predicted by fluorescent capillary electrophoresis (Fig. 4c,d). Clones #2 and #4, being homozygous mutants and whose indels were expected to cause a frameshift, displayed marked decrease in mRNA expression levels and complete ablation of protein expression. In contrast, the heterozygous mutants showed a less dramatic decrease in mRNA levels and slight decrease in protein levels. Interestingly, clones #1 and #6, which are homozygous mutant and compound heterozygous mutant respectively, expressed p53 comparable to parental HCT116 cells. This is likely due to in-frame indels harboured by these clones as supported by the discernible shift in protein size.

Lastly, we interrogated the efficacy of the fluorescent capillary electrophoresis technique to genotype a bi-allelic non-coding RNA gene, *MIR615*, specifically the region encoding miR615-3p. Due to its extremely short sequence, microRNA-encoding regions are expected to be easily knocked out. In accordance to this, we found that 70.8% of clones examined were mutated on at least one allele (Supplementary Table 1) and all the clones we analysed via qRT-PCR showed at least seven-fold decrease in miR615-3p expression level (Fig. 5b; and data not shown). Due to their complementary nature and mutual requirement for maturation, we expected that the mutation of miR615-3p will adversely affect the expression of miR615-5p as well. In agreement to that, we observed a similarly drastic decrease in miR615-5p expression in the three mutant clones examined (Fig. 5d). We also investigated the effects of miR615-3p mutation on a known target—*AKT2*^24^. All three clones showed significant increase in *AKT2* mRNA expression (Fig. 5e) and protein level (Fig. 5f) when compared to wildtype parental cells, indicating successful targeting of the *MIR615* gene.

In addition to HCT116 cells, we have successfully genotyped A2780/CP, RKO and H1 cells targeted using the same sgRNAs described above (data not shown). On top of that, we have also used the fluorescent capillary gel electrophoresis technique to successfully genotype HT1080 cells targeted using sgRNAs designed against human *TERT*, *WLS* and *NCOR2* genes (data not shown). Collectively, these results showed that the fluorescent capillary electrophoresis technique enables robust genotyping of CRISPR/Cas9-targeted clones, sensitive enough to differentiate between wildtype clones, heterozygous mutants, compound heterozygous mutants and homozygous mutants. More importantly, this technique accurately measures the number of nucleotide(s) inserted or deleted and thus effectively reports any shift in frame resulting in the formation of premature stop codon.

### Additional benefits of fluorescent capillary gel electrophoresis

As reported above, our targeting of exon 2 of *ATRX* gene with sgATRX-E2 did not yield detectable levels of cleavage in the SURVEYOR mutation detection assay (Fig. 2a). However, we were interested to find out whether the fluorescent capillary gel electrophoresis technique is able to circumvent the detection limitation of SURVEYOR mutation detection assay and is sensitive enough to detect mutants at this target site. We performed similar experiments as described above with sgATRX-E2 and identified mutants at a very low rate (3 out of 32; Supplementary Table 1; and Supplementary Figure S4a). We sequenced these clones and found that they indeed harboured indel mutations at the expected CRISPR/Cas9 cutting site (Supplementary Figure S4b). Hence, we conclude that fluorescence PCR-capillary gel electrophoresis is an efficient way of detecting CRISPR/Cas9-targeted mutants even when the efficacy of the sgRNA is very low. This technique, thus, possibly precludes the need for testing the efficacy of sgRNA prior to its use in targeting specific regions of the genome since the screening platform we describe here is capable of handling a large sample size.

A potential problem that one may encounter during handling of large number of clones is the cross-contamination between clones resulting in heterogeneity of cell population, especially during the colony picking process to array individual clones onto 96-well plates from 10-cm dishes. Due to its high sensitivity and resolving power, we anticipated the ability of fluorescent capillary gel electrophoresis to identify mixed population of cells. Indeed, we found an sgATRX-E2-targeted clone with an unusual peak pattern. As *ATRX* is a mono-allelic gene in HCT116 background, both wildtype and mutant clones are expected to display single peaks. However, clone #S6 displayed two peaks in its fluorescent capillary gel electrophoresis analysis—a smaller peak corresponding to wildtype sequence and a taller peak with deleted sequence (Supplementary Figure S5a). Sanger sequencing confirmed the heterogeneity of the cell population (Supplementary Figure S5b, top) and deletion of nucleotides in the mutant genome (Supplementary Figure S5b, bottom). As this deletion of 17 base pairs is expected to cause a frameshift, full-length ATRX protein is expected to be lowly expressed. However, we observed ATRX levels comparable to untargeted HCT116 cells in this clone (Supplementary Figure S5c). Hence, this shows that fluorescent capillary gel electrophoresis enables the identification of heterogeneous population of cells by the presence of aberrant peak pattern.

### Fluorescent capillary gel electrophoresis technique is multiplexable

An advantage of the CRISPR/Cas9 system over other programmable nucleases (ZFNs and TALENs) is its ability to simultaneously target multiple genes efficiently^19^. Therefore, we sought to test the robustness of the fluorescent capillary gel electrophoresis technique in multiplex detection of indel mutations in two different genes in the same clone. We co-transfected HCT116 cells with sgATRX-E4 and sgTP53-E4.2 expression plasmids and pCas9-2A-GFP plasmid and sorted for GFP-positive cells. Individual clones were arrayed on a 96-well plate and subjected to fluorescent capillary gel electrophoresis analysis for both the targeted genes. About 18% of clones examined displayed double-gene targeting (Supplementary Table 1). We sequenced some of these clones and found excellent agreement between the fluorescent capillary gel electrophoresis results and the sequencing results. Figure 6a,b show the results of two representative clones. We performed RT-PCR and western blot analyses and found that the results from these experiments corroborate the double knockout status of these two clones (Fig. 6c,d). These results show that the fluorescent capillary gel electrophoresis technique supports multiplex genome targeting via CRISPR/Cas9 system, enabling fast and accurate screening of multiply targeted cells.

## Discussion

Inherent technical limitations and high cost of present genotyping methods render these techniques not ideal for high-throughput detection of CRISPR/Cas9-induced indel mutants in targeted cells. Whilst there have been various recent reports on the improvements of these current genotyping methods, many of them tend to focus on nuclease-mediated mutations at the cell population level and are not very informative in that they do not report the occurrence of frameshift in the sequence of individual clones. For example, the high resolution melting analysis (HRMA) protocol described by Thomas and colleagues^9^ is able to distinguish distinct mutant alleles based on their intricate DNA melting profiles. However, this technique is not sensitive enough to report the presence or absence of frameshift in these alleles and it thus necessitates a second round of screening for a knockout clone. Another example of an advancement in the field is the introduction of TIDE (Tracking of Indels by DEcomposition) by Brinkman and co-workers^25^ which uses a decomposition algorithm to analyse capillary sequencing traces to determine the major induced mutations in a mixed population of cells. This method is extremely useful for the assessment of the cutting efficiency of programmable nucleases but is not compatible for high-throughput screening due to its high cost engendered by the sequencing step.

Recently, yet another two more genotyping techniques have been described. Yu and colleagues reported a PCR-based method which utilizes a primer overlapping the putative indel site to determine the presence of CRISPR/Cas9-induced mutations at these sites^26^. In addition, Zhu and his team introduced a polyacrylamide gel electrophoresis (PAGE)-based technique to distinguish wildtype and mutant sequences on the basis of differential mobility rate of homoduplex and heteroduplex DNA formed from the annealing of denatured DNA amplicons^27^. Whilst these techniques are advantageous especially in circumventing certain drawbacks inherent in the other genotyping techniques mentioned above, they too suffer from a certain drawback common to most other genotyping techniques: they do not report the presence or absence of frameshift mediated by CRISPR/Cas9 DNA cleavage, which the technique we have described herein is capable of.

Taking all these factors into consideration, we therefore sought to assess the possibility of employing fluorescence capillary electrophoresis for the very purpose of genotyping CRISPR/Cas9-induced mutants. This technique has previously been used to genotype populations of nuclease-mediated cells mainly to determine the cleavage efficacy of the nuclease system^22^,^28^. Here, we show that this time- and cost-saving technique is able to efficiently and accurately detect indel mutations in CRISPR/Cas9-targeted sequences in single clones and hence effectively identify knockout clones. We targeted three genes on three different chromosomes and found that the fluorescent capillary electrophoresis technique could genotype the targeted clones highly accurately.

In addition to its amenability for high-throughput screening, the fluorescent capillary electrophoresis technique has several other advantages. Firstly, this technique reports accurately the number of nucleotides inserted or deleted at the non-homologous end joining repair-mediated DSB site. This essentially obviates the need for sequencing analysis to determine the occurrence of a frameshift which may potentiate the ablation of expression of the targeted gene. This advantage stems from the high sensitivity and resolving power of this technique and is evident in its ability to identify mutants with indel of just one base pair. Secondly, this technique is able to support multiplex targeting of genes. As a result, users stand to save time and cost by performing genotyping analysis in a single 96-well plate using different primers for the various targeted regions. Thirdly, this fluorescent capillary electrophoresis protocol allows for the identification of erroneous heterogeneous cell populations which is crucial when studying the effects of a knocked out gene where homogeneity of clones is key.

Two factors are important for this technique’s amenability for high-throughput screening. First, the use of our direct lysis buffer, Direct-Lyse, allows for fast and reproducible extraction of crude genomic DNA straight from cells in culture. Furthermore, the resultant lysates are stable for months if kept at lower than −20 °C. We found that the Direct-Lyse buffer is comparably efficacious as other commercially available direct lysis buffer and that it is more cost-effective in the long term. Secondly, as compared to Sanger sequencing which is currently the most widely used method of genotyping targeted clones, the use of fluorescent capillary electrophoresis is markedly cheaper and therefore effectively scalable. The cost of genotyping a targeted clone using Sanger sequencing is approximately US$4-8 per reaction, including the DNA purification step and the actual sequencing reaction; and depending on the method of purification and the number of samples handled at a time (throughput). In comparison, the capillary gel electrophoresis technique described herein costs about US$3-4 per reaction. This amounts to a cost savings of between 25% and 50%. In addition, the fluorescent PCR-capillary gel electrophoresis technique requires less than half the time to obtain the genotyping results as compared to Sanger sequencing which typically requires third-party services.

There are several caveats associated with the fluorescent capillary gel electrophoresis technique we have described. For one, this technique is not adequately accurate at reporting mutations with indels of more than 30 base pairs. It tends to overestimate these mutations. However, we find that this may not necessarily be a problem because most of the mutations we have seen in our study involve indels shorter than that threshold. Another limitation of this technique is its inability to detect base substitutions or single nucleotide polymorphisms (SNPs) in the targeted genome which may result in the formation of missense or nonsense mutations. Whilst this type of mutation may result in the decrement or even abolition of gene expression, it has been reported that non-homologous end joining-mediated repair of DSBs rarely results in the substitution of bases in the nucleotides found at the break sites^11^. Thirdly, our protocol requires the use of a genetic analyser equipment and analysis software which may not be readily available. However, we found that this part of the experiment can be outsourced to companies which provides sequencing services and at a cost comparable to when done on our own. Lastly, since our targeting strategy involves the transfection of two separate plasmids for the two components of the CRISPR/Cas9 genome-targeting system—Cas9 and sgRNA—the targeting efficiencies we report are not very high. We expect that the use of a bicistronic expression plasmid containing both CRISPR components will greatly improve targeting efficiency, coupled with the enrichment step of sorting for GFP-positive cells from the expression of Cas9-2A-GFP cassette, as shown previously^29^.

Thus, we have described a high-throughput method of genotyping CRISPR/Cas9-mediated mutation of human cells using fluorescent capillary gel electrophoresis. We anticipate this technique to be applicable to a wide range of targets and model systems. Moreover, with its amenability for high-throughput screening, fast and efficient genotyping of mutants or knockout clones may no longer be a bottleneck for gene targeting studies.

## Methods

### Reagents

All oligonucleotides used in our study were procured from Sigma-Aldrich Co. and Life Technologies and are listed in Supplementary Table 2. Oligonucleotides labelled with 6-FAM or HEX were covalently bonded with the fluorophore at the 5′ end. The following antibodies were used in our study: rabbit polyclonal anti-ATRX antibody from Santa Cruz Biotechnology (sc-15408); mouse monoclonal anti-p53 antibody from Santa Cruz Biotechnology (sc-126); mouse monoclonal anti-β-actin antibody from Sigma-Aldrich Co. (A1978); rabbit monoclonal anti-Akt2 antibody from Cell Signaling Technology (#3063); sheep anti-mouse HRP-conjugated secondary antibody from Jackson ImmunoResearch; and goat anti-rabbit HRP-conjugated secondary antibody from Jackson ImmunoResearch. All antibodies were diluted in 5% milk in TBST buffer.

### Selecting sgRNA targets

Exon 2 and 4 of human *ATRX* gene and miR615-3p-encoding region of *MIR615* were subjected to potential sgRNA target search using the online software created by Feng Zhang’s group (http://tools.genome-engineering.org)^19^. The very top hits were chosen and used for the experiments hence described. The nucleotide sequence of these sgRNA targets are given in Fig. 2 and Supplementary Table 3.

### Plasmid construction

The pCas9-GFP plasmid was obtained from Addgene (#44719). Both *TP53* sgRNA (sgTP53-E4.1 and -E4.2) expression plasmids were gifts from Voorhoeve’s laboratory. *ATRX* sgRNAs (sgATRX-E2 and -E4) and *MIR615* sgRNA (sgMIR615-3p-T1, -T2, -T3 and -T4) expression plasmids were constructed by cloning U6 promoter-sgRNA-TTT [(as used by Mali *et al*.^30^] into pBluescript SK (-) vector (see Supplementary Figure S1 for schematic). Briefly, the sgRNA expression cassettes were divided into twelve overlapping oligonucleotides and assembled into a contiguous DNA fragment via two rounds of PCR. The inserts and pBSK(−) vector were then digested with SpeI and PstI (Hoffmann-La Roche), resolved on a 1% agarose gel, purified from excised gel using Wizard SV Gel and PCR Clean-Up System (Promega) and ligated using T4 DNA Ligase (New England Biolabs). DH5α cells were transformed with the ligation product and plated on Luria Bertani agar supplemented with ampicillin, IPTG and X-gal. Individual white colonies were picked and cultured, and corresponding plasmids extracted using Wizard Plus SV Minipreps DNA Purification System (Promega) and sequenced to ensure correct insert sequence.

### Selecting lysis conditions

Comparisons of direct lysis efficiency between 1× and 0.5× Direct-Lyse buffer, and between 0.5× Direct-Lyse buffer and sodium hydroxide lysis buffer were performed using H1 human embryonic stem cell line and A2780/CP human ovarian carcinoma cell line, respectively. Monolayer of cells on a 96-well plate were trypsinised using 25 μl of 0.25% trypsin/EDTA (without phenol red) at 37 °C for 10–15 minutes and 5 μl of the cell suspension was added to 10 μl of one of the lysis buffer: 1× Direct-Lyse buffer (20 mM Tris pH 8.0, 5 mM EDTA, 0.4 M NaCl, 0.3% SDS, 0.6% Tween-20); 0.5× Direct-Lyse buffer (10 mM Tris pH 8.0, 2.5 mM EDTA, 0.2 M NaCl, 0.15% SDS, 0.3% Tween-20); or 2× sodium hydroxide lysis buffer (50 mM NaOH, 0.4 mM EDTA). Direct-Lyse lysates were subjected to a series of thermal cycling as follows: 65 °C for 30 s, 8 °C for 30 s, 65 °C for 1.5 min, 97 °C for 3 min, 8 °C for 1 min, 65 °C for 3 min, 97 °C for 1 min, 65 °C for 1 min, and 80 °C for 10 min. Sodium hydroxide lysates were heated at 100 °C for 20–30 min after which 10 μl of 40 mM Tris-HCl (pH 5.0) was added to neutralize the pH. Finally, each of these lysates was diluted with 40 μl water before being subjected to PCR analysis. To this end, 3 μl of the diluted lysates together with Platinum Taq DNA polymerase (Life Technologies) were used to amplify various genomic regions using primers listed in Supplementary Table 2 following manufacturer’s recommendation. Amplicons were resolved on 1% agarose gel, stained with ethidium bromide and imaged using ChemiDoc XRS System (Bio-Rad).

### Cell culture and transfection conditions

All CRISPR/Cas9 targeting experiments were performed with HCT116 human colorectal carcinoma cell line or HEK293 human embryonic kidney cell line (both from ATCC). Cells were maintained in normal growth media (DMEM containing 10% FBS and 1% penicillin/streptomycin) except prior to and during DNA transfection. For targeting of individual genes, cells were seeded at 2 × 10^5^ cells per well of a 6-well plate in antibiotic-free media (DMEM with 10% FBS) and co-transfected with pCas9-GFP and one (for single gene targeting) or two (for multiplexed targeting) of the sgRNA expression plasmid or empty vector [pBluescript SK (−)] using Lipofectamine 2000 (Life Technologies) as per manufacturer’s recommendation. We co-transfected the cells with 0.5 μg pCas9-GFP plasmid and 3.5 μg sgRNA expression plasmid using 10 μl Lipofectamine 2000 transfection reagent for about 16 hours and changed to fresh media thereafter. For simultaneous targeting of *TP53* and *ATRX* genes, cells were seeded at 2 × 10^6^ cells per 10-cm dish and co-transfected with 1 μg pCas9-GFP plasmid and 11.5 μg each of pBSK-sgTP53-E4.2 and pBSK-sgATRX-E4 plasmids using 60 μl Lipofectamine 2000 transfection reagent.

### Fluorescence-activated cell sorting

HCT116 cells that were used for SURVEYOR assay and fluorescent capillary gel electrophoresis were subjected to cell sorting. Two days after transient transfection, cells were sorted using FACSAria III (BD Biosciences). GFP-positive clones were collected and plated accordingly. For purpose of performing SURVEYOR assay, 1 × 10^4^ cells were plated on 6-well plate and maintained until confluent; whereas for isolation of individual clones, cells were plated on 10-cm dish at 500 to 2,500 cells per dish until individual colonies were visible.

### SURVEYOR mutation detection assay

HEK293 and HCT116 cells were used to perform SURVEYOR mutation detection assay with *ATRX* and *TP53* sgRNAs, respectively. The latter were subjected to FACS to enrich for GFP-positive cells prior to SURVEYOR assay, whilst crude transfected cells were used for the former. When cells reached confluence, they were harvested and their genomic DNA extracted using Gentra Puregene Cell Kit (QIAGEN). 100 ng of genomic DNA was used to amplify the regions spanning CRISPR/Cas9 cut sites using specific primers (see Supplementary Table 2) and Platinum Taq DNA Polymerase High Fidelity (Life Technologies) as per manufacturer’s instruction. Control C and G fragments (Transgenomic, Inc.) were also amplified in parallel. The PCR products were purified using QIAquick PCR Purification Kit (QIAGEN) and diluted to 100 ng/μl. 2.15 μg of the purified PCR product were added to 1× Platinum Taq DNA Polymerase High Fidelity buffer (Life Technologies) and 2 mM magnesium sulphate, and subjected to a denaturation and re-annealing program to facilitate the formation of mismatched fragments: 95 °C for 10 minutes, ramping from 95 °C to 85 °C at 2 °C/s, 85 °C for 1 minute, ramping from 85 °C to 75 °C at 0.3 °C/s, 75 °C for 1 minute, ramping from 75 °C to 65 °C at 0.3 °C/s, 65 °C for 1 minute, ramping from 65 °C to 55 °C at 0.3 °C/s, 55 °C for 1 minute, ramping from 55 °C to 45 °C at 0.3 °C/s, 45 °C for 1 minute, ramping from 45 °C to 35 °C at 0.3 °C/s, 35 °C for 1 minute, ramping from 35 °C to 25 °C at 0.3 °C/s, and 25 °C for 1 minute. Next, 15 μl of the re-annealed DNA were added to 0.5 μl of Enhancer S and 0.5 μl of Nuclease S (Transgenomic, Inc.) and incubated at 42 °C for an hour. The digestion products were resolved on a 10% polyacrylamide/TBE gel and stained with ethidium bromide for 15 minutes. The gels were visualized using ChemiDoc XRS System (Bio-Rad Laboratories, Inc.) and the fragments quantified using ImageJ. Indels were calculated using the following formula, where *a* is the intensity of the undigested fragment, and *b* and *c* are the intensities of the cleavage products.


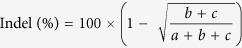


### Direct lysis of cultured cells

Around 10–12 days after plating of GFP-positive FACS-sorted cells onto 10-cm dish, individual colonies were picked and transferred to 96-well plates and maintained. When cells in the 96-well plate reached about 80% confluence, they were subjected to direct lysis using 0.5× Direct-Lyse buffer (10 mM Tris pH 8.0, 2.5 mM EDTA, 0.2 M NaCl, 0.15% SDS, 0.3% Tween-20). Growth media was removed from the wells and cells were trypsinised with 0.05% trypsin-EDTA (without phenol red) for 7 minutes. About 5 μl of the cell suspension were added to 10 μl of Direct-Lyse lysis buffer in a 96-well PCR plate. The cells were then subjected to a series of heating and cooling to ensure complete lysis: 65 °C for 30s, 8 °C for 30s, 65 °C for 1.5 min, 97 °C for 3 min, 8 °C for 1 min, 65 °C for 3 min, 97 °C for 1 min, 65 °C for 1 min, and 80 °C for 10 min. The lysates were then diluted with 40 μl of water and subjected to diagnostic PCR analyses.

### Fluorescent PCR

Diluted lysates were used to amplify regions spanning CRISPR/Cas9 targeting sequence in a high-throughput manner on a 96-well plate to identify clones harbouring indel mutations. Platinum Taq DNA Polymerase (Life Technologies) was used for this purpose, together with primer pairs with 5′ modification on the forward primers (Supplementary Table 2). Lysates of parental wildtype HCT116 cells were subjected to PCR amplification with HEX-labelled forward primers, whereas those of the CRISPR/Cas9-targeted clones were performed with 6-FAM-labeled primers. A part of these labelled amplicons were then resolved on a 1% agarose gel to estimate their relative amounts and the remaining were subsequently diluted to roughly standardize their concentration, before being subjected to capillary gel electrophoresis. Typically, the amplicons were diluted 30× with water and those which showed lower amounts were diluted by a lower factor.

### Capillary gel electrophoresis

Diluted fluorescent PCR amplicons from wildtype and targeted cells were mixed 1:1 and 1 μl of the mixture was added to 8.7 μl Hi-Di Formamide and 0.3 μl GeneScan 500 LIZ dye Size Standard (Life Technologies) on a 96-well plate. As per manufacturer’s instruction, the resultant mixture was heated at 95 °C for 3 minutes and subsequently cooled on ice for 3 minutes. These samples were resolved via capillary gel electrophoresis on a 3500xL Genetic Analyzer (Life Technologies). The details of the instrument protocol and the size-calling protocol used are as follow—application type: fragment; capillary length: 50 cm; polymer: POP7; dye set: G5; run voltage: 19.5 kV; pre-run voltage: 15 kV; injection voltage: 1.6 kV; run time: 1330 s; pre-run time: 180 s; injection time: 15 s; data delay: 1 s; size standard: GS500(-250)LIZ; size-caller: SizeCaller v1.10. Results were analysed using Gene Mapper 5 software (Life Technologies). Insertions or deletions of nucleotides at corresponding CRISPR/Cas9 cleavage sites were estimated by calculating the difference in fragment sizes as provided by the analysis software.

### Sequencing of targeted clones

Clones predicted to contain indel mutations due to the CRISPR/Cas9 targeting were isolated and the regions spanning these mutations were amplified via PCR using primers listed in Supplementary Table 2 and Platinum Taq DNA Polymerase High Fidelity (Life Technologies) following manufacturer’s recommendation. The resultant amplicons were sent for sequencing with their respective 5′ PCR primers. For compound heterozygous mutants, we cloned the PCR amplicons into pCRII-Blunt-TOPO vector using the Zero Blunt TOPO PCR Cloning Kit (Life Technologies) and transformed DH5α cells with the ligation product. Transformants were picked and cultured overnight and their plasmids were extracted using Wizard Plus SV Minipreps DNA Purification System (Promega) and sent for sequencing with their respective 5′ PCR primers.

### Quantitative RT-PCR

Clones were cultured on 6-well plates and harvested when they reached confluence. Total RNA was extracted from the clones using NucleoSpin RNA kit (Macherey-Nagel GmbH & Co.) and their concentration measured using Nanodrop 2000 (Thermo Fisher Scientific Inc.). The RNA samples were diluted to 100 ng/μl and 300 ng of each were used to perform quantitative RT-PCR using KAPA SYBR FAST Bio-Rad iCycler (Kapa Biosystems) and CFX96 Touch Real-Time PCR Detection System (Bio-Rad Laboratories, Inc.) in technical triplicates and in biological duplicates. Relative mRNA expression of individual clones was calculated using the comparative C_T_ (ΔΔC_T_) method, normalized to β-actin and relative to wildtype parental cells. Mean fold change, standard deviation, and p-value (using one-tailed t-test) were calculated using Microsoft Excel.

### Western blot analyses

Clones were cultured on 10-cm dish and harvested when they reached confluence. Cells were lysed with Lysis 250 Buffer (50 mM Tris-HCl pH 7.4, 250 mM NaCl, 5 mM EDTA pH 8.0, 0.1% NP-40, 50 mM NaF) supplemented with Complete Protease Inhibitor (Hoffmann-La Roche), by a series of freeze-thaw cycles. Crude lysates were centrifuged to remove cell debris and the clarified lysates were used directly for immunoblotting (*TP53* clones, and *MIR615-3p* clones probing for Akt2) or for immunoprecipitation prior to blotting (*ATRX* clones). About 75 μg and 8 mg of total lysates were used for immunoblotting and immunoprecipitation, respectively. To the lysates of *ATRX*-targeted clones, 1 μg of anti-ATRX antibody (sc-15408; Santa Cruz Biotechnology, Inc.) was added and incubated for an hour before the addition of ~50 μl GammaBind G Sepharose beads (GE Healthcare). The immunoprecipitation mixtures were incubated overnight at 4 °C with constant rotation and the protein-bound beads were subsequently washed three times with Lysis 250 Buffer. Next, the beads were heated at 95 °C for 10 minutes and centrifuged at top speed for a minute before the resultant supernatants were resolved on a 6% polyacrylamide gel. Similarly for *TP53*- and MIR615-3p-targeted clones, samples were mixed with SDS loading dye and heated at 95 °C for 10 minutes and centrifuged at top speed for a minute and loaded onto a 10% polyacrylamide gel. Resolved proteins were transferred onto PVDF membranes at 400 mA for 1.5 to 2 hours. The membranes were blocked with milk (5% in TBST) for an hour at room temperature before incubated overnight with primary antibody. They were then washed with TBST for at least 30 minutes and incubated at room temperature with secondary antibody for an hour. Lastly, the membranes were washed with TBST and subjected to ECL detection. Anti-ATRX antibody (Santa Cruz) was diluted 1:200; anti-p53 antibody (Abcam) was diluted 1:1,000; anti-Akt2 antibody (Cell Signaling) was diluted 1:1,000; and anti-β-actin antibody was diluted 1:40,000. Sheep anti-mouse HRP-conjugated antibody (1:20,000; Jackson ImmunoResearch) and goat anti-rabbit HRP-conjugated antibody (1:20,000; Jackson ImmunoResearch) were used as secondary antibodies.

## Additional Information

**How to cite this article**: Ramlee, M. K. *et al*. High-throughput genotyping of CRISPR/Cas9-mediated mutants using fluorescent PCR-capillary gel electrophoresis. *Sci. Rep*. **5**, 15587; doi: 10.1038/srep15587 (2015).

## Supplementary Material

Supplementary Information
