# Supplementary material for: High-throughput genotyping of CRISPR/Cas9-mediated mutants using fluorescent PCR-capillary gel electrophoresis
Source: Sci Rep. 2015 Oct 26;5:15587. doi: 10.1038/srep15587 (PMC4620477; doi:10.1038/srep15587)
Supplement: Supplementary Information [file srep15587-s1.pdf]

High-throughput genotyping of CRISPR/Cas9-mediated mutants using fluorescent PCR-capillary gel electrophoresis

Muhammad Khairul RAMLEE, Tingdong YAN, Alice M. S. CHEUNG, Charles CHUAH, Shang LI

Figure Legends

Supplementary Figure S1. Schematic of engineering of sgRNA expression cassette. (a) sgRNA expression cassette sequence (U6 promoter-sgRNA-TTT; given in grey box) were divided into 12 overlapping oligonucleotides which were pieced together via two rounds of PCR. (b) Sequences of the 12 oligonucleotides used in (a).

Supplementary Figure S2. Testing direct lysis conditions. (a) Crude genomic DNA extracted from H1 cells using 1x and 0.5x Direct-Lyse buffer were subjected to PCR analysis to determine the optimum direct lysis conditions. (b) Comparison between lysis efficiency of 0.5x Direct-Lyse buffer and sodium hydroxide lysis buffer in A2780/CP cells. (c) HCT116 cells were lysed using 0.5x Direct-Lyse buffer and the crude genomic DNA were subjected to PCR analysis using the primers indicated below the lanes to test the compatibility of the buffer for high throughput screening of CRISPR/Cas9-targeted clones. M: DNA ladder.

Supplementary Figure S3. Additional *ATRX*- and *TP53*-targeted clones genotyped via fluorescent PCR-capillary gel electrophoresis. The genotype of HCT116 cells targeted using sgATRX-E4 and sgTP53-E4.2 were determined via fluorescent PCR-capillary gel electrophoresis (a and c) and verified via Sanger sequencing (b and d). All symbols and representations are identical to those in Fig. 3.

Supplementary Figure S4. Fluorescent PCR-capillary gel electrophoresis technique is able to detect mutants targeted using low-efficiency sgRNA. HCT116 cells were targeted using sgATRX-E2 and individual clones were genotyped via fluorescent PCR-capillary gel electrophoresis. Two clones were predicted to harbor single-base insertion (a) and this was confirmed via Sanger sequencing (b). All symbols and representations are identical to those in Fig. 3.

Supplementary Figure S5. Fluorescent PCR-capillary gel electrophoresis technique is able to detect heterogeneity of cell population. An HCT116 clone targeted using sgATRX-E2 showed unexpected peak pattern (i.e. two peaks whereas ATRX is mono-allelic in HCT116 background) in fluorescent PCR-capillary gel electrophoresis assay (a) indicating presence of two population of cells in the sample. Sanger sequencing (b) and Western blot (c) analyses confirmed heterogeneity of cell population.

Supplementary Table 1. Genotype of CRISPR/Cas9-Mediated Gene Targeted Clones via Fluorescent Capillary Gel Electrophoresis.

Supplementary Table 2. List of oligonucleotides used in study.

Supplementary Table 3. Spacer sequence of sgRNAs.

Supplementary Figure S1

a

```
TTTTACTAGTTGTACAAAAAAGCAGGCTTTAAAGGAACCAATTCAGTCGACTGGATCCGGTACCAAGGTCGGGCAGGAAGAGGGCCTATTTCCCATGATTCCT
TCATATTTGCATATACGATACAAGGCTGTTAGAGAGATAATTAGAATTAATTTGACTGTAAACACAAAGATATTAGTACAAAATACGTGACGTAGAAAGTAAT
AATTTCTTGGGTAGTTTGCAGTTTTAAAATTATGTTTTAAAATGGACTATCATATGCTTACCGTAACTTGAAAGTATTTCGATTTCTTGGCTTTATATATCTT
GTGGAAAGGACGAAACACC(G)NNNNNNNNNNNNNNNNNNNNGTTTTAGAGCTAGAAATAGCAAGTTAAAATAAGGCTAGTCCGTTATCAACTTGAAAAAGTG
GCACCGAGTCGGTGCTTTTTTTCTAGACCCAGCTTTCTTGTACAAAGTTGGCATTACTGCAGTTTT
```

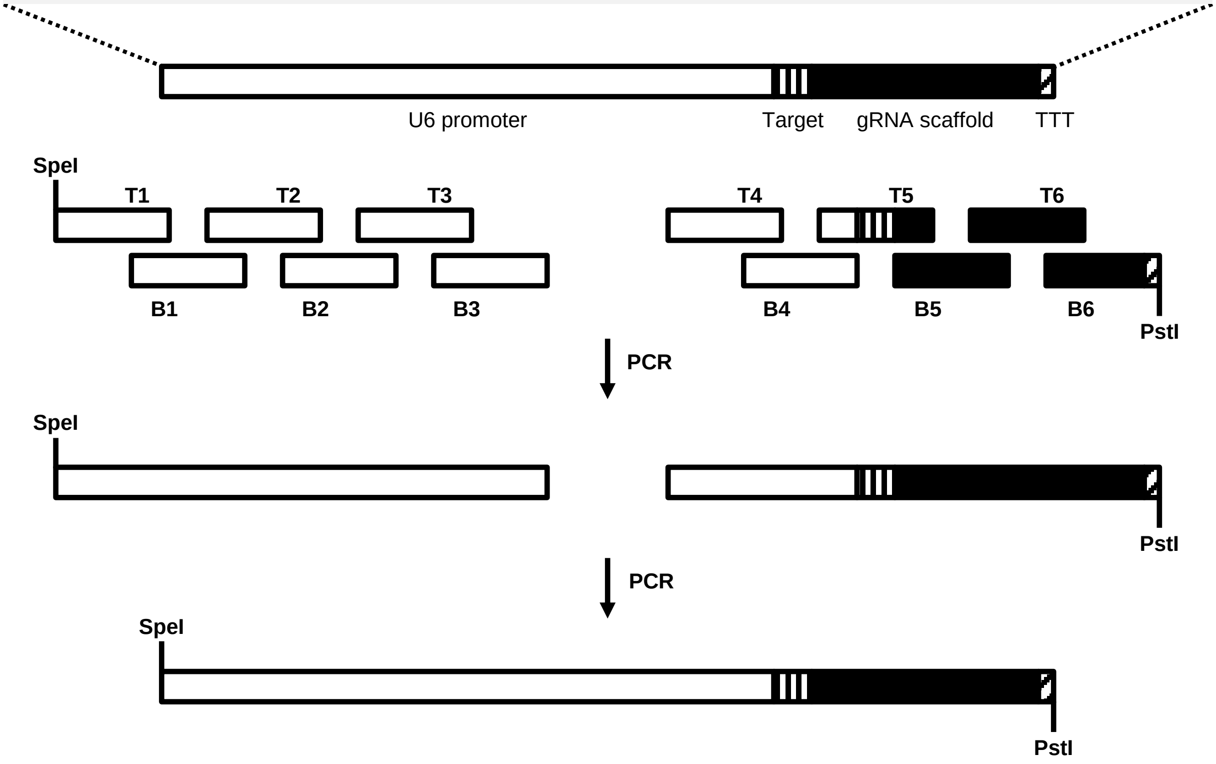

b

Oligonucleotide	Sequence
Top1	TTTTACTAGTTGTACAAAAAAGCAGGCTTTAAAGGAACCAATTCAGTCGACTGGATCCGG
Top2	GGAAGAGGGCCTATTTCCCATGATTCCTTCATATTTGCATATACGATACAAGGCTGTTAG
Top3	TAATTTGACTGTAAACACAAAGATATTAGTACAAAATACGTGACGTAGAAAGTAATAATT
Top4	AGTTTTAAAATTATGTTTTAAAATGGACTATCATATGCTTACCGTAACTTGAAAGTATTT
Top5	TATATCTTGTGGAAAGGACGAAACACC(G)N_{20}GTTTTAGAGCTAGAAATAGCAA
Top6	TAAGGCTAGTCCGTTATCAACTTGAAAAAGTGGCACCGAGTCGGTGCTTTTTTTCTAGAC
Bottom1	TCATGGGAAATAGGCCCTCTTCCTGCCCGACCTTGGTACCGGATCCAGTCGACTGAATTG
Bottom2	TCTTTGTGTTTACAGTCAAATTAATTCTAATTATCTCTCTAACAGCCTTGTATCGTATAT
Bottom3	TTTTAAAACATAATTTTAAAACTGCAAACTACCCAAGAAATTATTACTTTCTACGTCACG
Bottom4	TTCGTCCTTTCCACAAGATATATAAAGCCAAGAAATCGAAATACTTTCAAGTTACGGTAA
Bottom5	AGTTGATAACGGACTAGCCTTATTTTAACTTGCTATTTCTAGCTCTAAAAC
Bottom6	AAAAGCTAGCTAATGCCAACTTTGTACAAGAAAGCTGGGTCTAGAAAAAAAGCACCGACT

Supplementary Figure S2

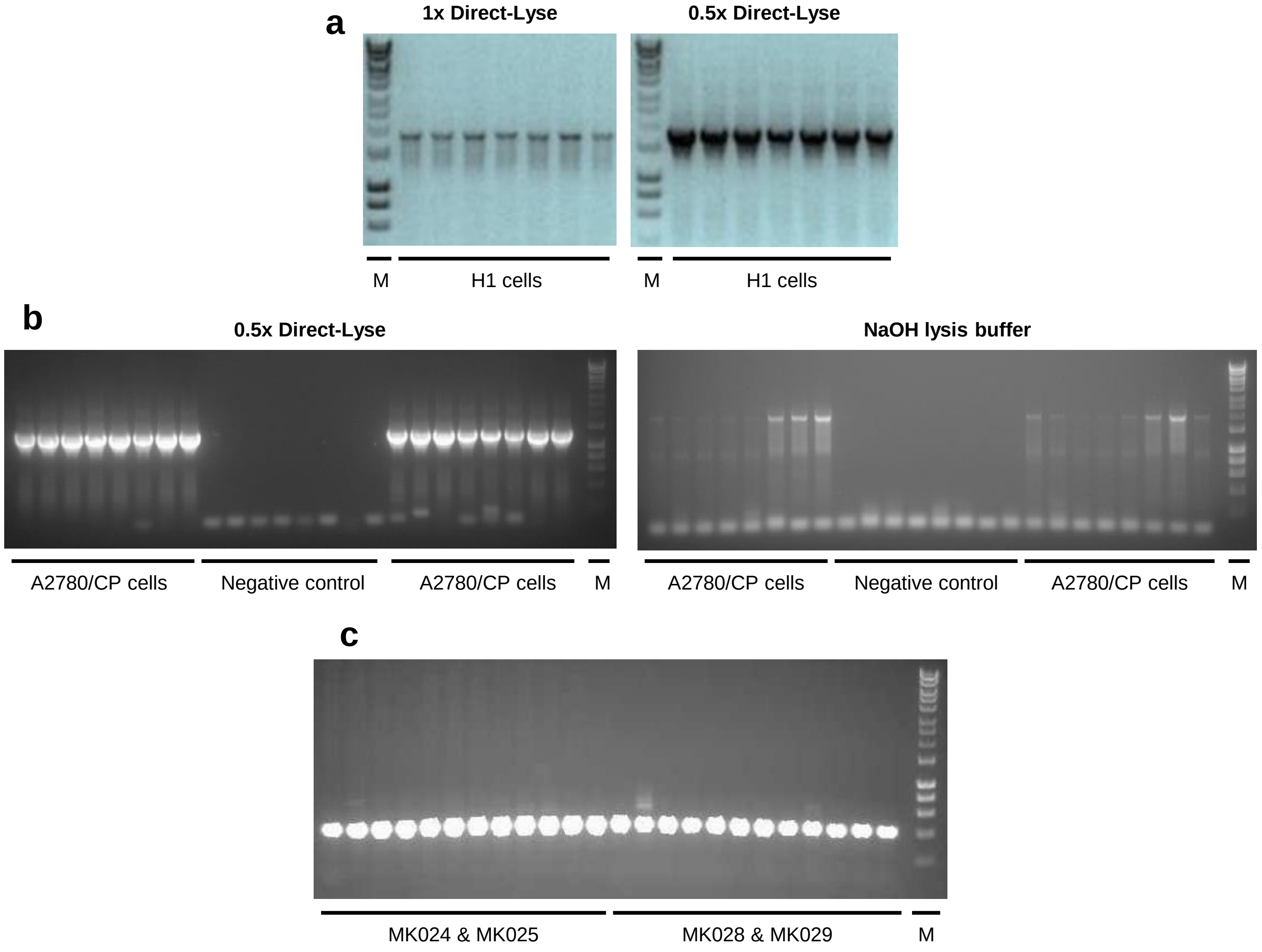

Supplementary Figure S3

a

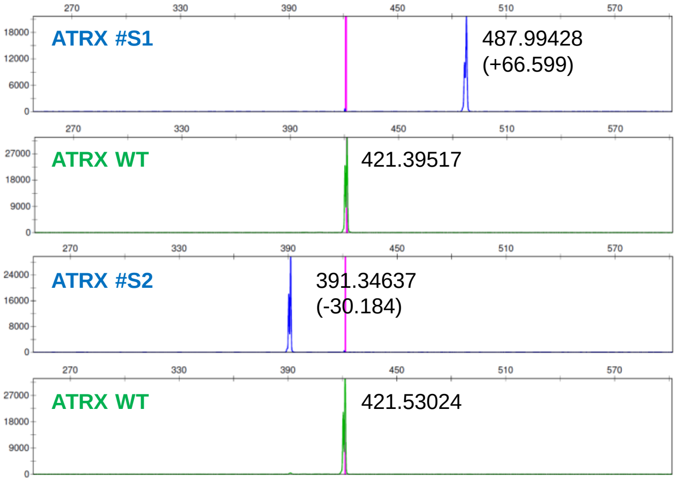

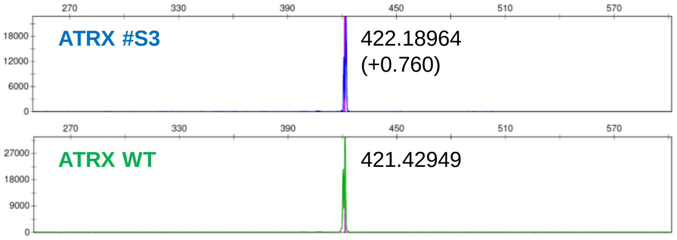

b

```
ATRX    AACTAGCTCTTCAGAAAAATCCAAGTCTTCAGGATCGTCACGA   WT

#S1     GCCGCGCGCTGCCTTCGCCCCGTGCCCCGCTCCGCCGCCGCCT
        CGCGCCGCCCGCCCCGGCTCTGACTG
        AACTAGCTCTTCAGAAAAATCCAAGTCTTCAGGATCGTCACGA   +69bp

#S2     AACTAGCTC------------------------------ACGA   -30bp

#S3                                 C
        AACTAGCTCTTCAGAAAAATCCAAGTCTTCAGGATCGTCACGA   +1bp
```

c

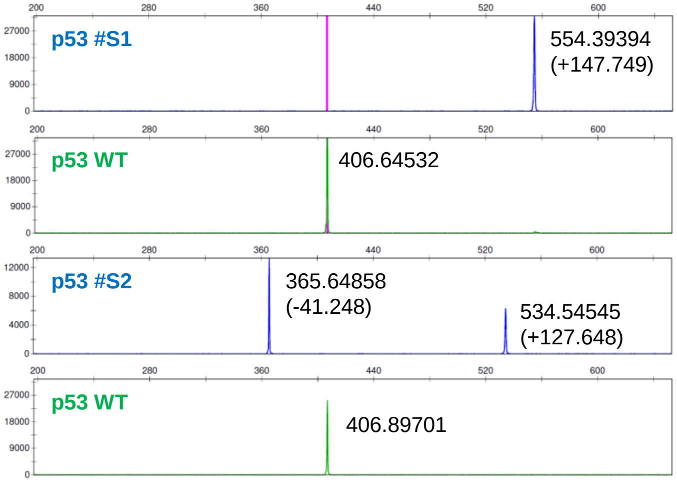

d

```
TP53    TTCTGTCCC/29bp/GTTTCCGTCTGGGCTTCTTGCATTCTGG   WT

#S1     CAATGCCCAGCTTTCCTTCGCAACTCGAGTGGAAGATTGGACT
        TGCCTGGGCCAACACAAGAGGCATCCTGATGAACTTCAGGGTC
        AGCTTGCCGTAGGTGGCATCGCCCTCGCCCTCGCCGGACACGC
        TGAACTTGTGGCC
        TTCTGTCCC/29bp/GTTTCCGTCTGGGCTTCTTGCATTCTGG   +142bp
        CAATGCCCAGCTTTCCTTCGCAACTCGAGTGGAAGATTGGACT
        TGCCTGGGCCAACACAAGAGGCATCCTGATGAACTTCAGGGTC
        AGCTTGCCGTAGGTGGCATCGCCCTCGCCCTCGCCGGACACGC
        TGAACTTGTGGCC
        TTCTGTCCC/29bp/GTTTCCGTCTGGGCTTCTTGCATTCTGG   +142bp

#S2     CGAGCTGGTCTCGGCAAGCGGTGCCCAACATGGGGGCTTGAAC
        CCGGGTCGAAGGGTCGCCCCGAAGCGACAGTTGGAGAACGCGG
        AACTACGCTGGGGAACACCCGAGTACTCTTAAGCAATCCCTGT
        GGTGAGTAAGAAGG
        TTCTGTCCC/29bp/GTTTCCG-------------------GG   -19bp; +143bp
                AGAAC
        TTCTGTCCC/----/------------------TGCATTCTGG   -47bp; +5bp
```

Supplementary Figure S4

a

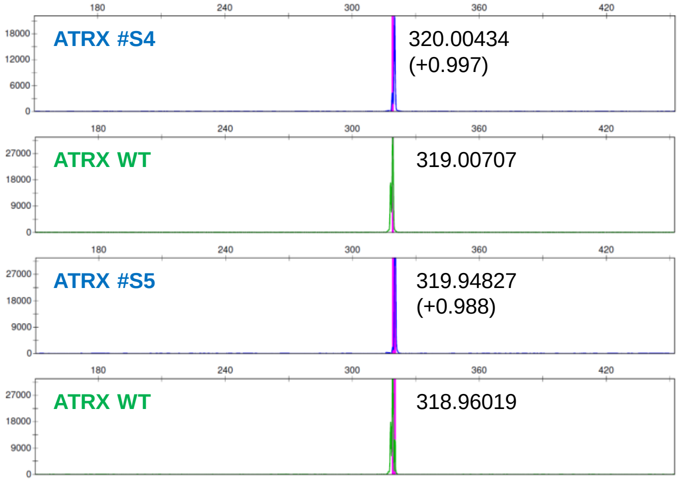

b

ATRX	CTGAAGAAACAAGTTCTCCTCCACGACTTGCAATGAATCAAAA	WT
#S4	C<br>CTGAAGAAACAAGTTCTCCTCCACGACTTGCAATGAATCAAAA	+1bp
#S5	T<br>CTGAAGAAACAAGTTCTCCTCCACGACTTGCAATGAATCAAAA	+1bp

Supplementary Figure S5

a

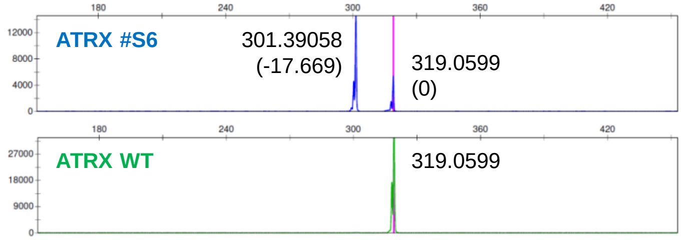

b

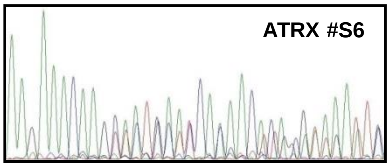

ATRX	CTGAAGAAACAAGTTCTCCTCCACGACTTGCAATGAATCAAAA	**WT**
**#S6**	CTGAAGAAACAAG-----------------CAATGAATCAAAA	-17bp

c

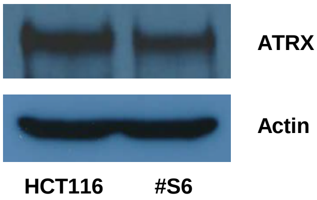

Supplementary Table 1. Genotype of CRISPR/Cas9-Mediated Gene Targeted Clones via Fluorescent Capillary Gel Electrophoresis

			TP53 mutant alleles per clone				*MIR615* mutant alleles per clone				*ATRX* mutant & *TP53* bi-allelic mutant clones
				2				2			
Gene targeted	sgRNA	*ATRX* mutant clones	1	Compound heterozygotes	Homozygotes	Total bi-allelic mutant clones	1	Compound heterozygotes	Homozygotes	Total bi-allelic mutant clones	
ATRX	sgATRX-E2	3/32 (9.4%)	-	-	-	-	-	-	-	-	-
	sgATRX-E4	17/26 (65.4%)	-	-	-	-	-	-	-	-	-
TP53	sgTP53-E4.1	-	2/43 (4.7%)	15/43 (34.9%)	17/43 (39.5%)	32/43 (74.4%)	-	-	-	-	-
	sgTP53-E4.2	-	2/38 (5.3%)	13/38 (34.2%)	17/38 (44.7%)	30/38 (78.9%)	-	-	-	-	-
MIR615	sgMIR615-T1 to -T4	-	-	-	-	-	7/48 (14.6%)	12/48 (25%)	15/48 (31.3%)	27/48 (56.3%)	-
ATRX & *TP53*	sgATRX-E4 & sgTP53-E4.2	-	-	-	-	-	-	-	-	-	7/39 (17.9%)

Supplementary Table 2. List of oligonucleotides used in study

Oligonucleotide	Sequence	Direction	Purpose
MK024	TGGTCCTCTGACTGCTCTT	Forward	For SURVEYOR assay and sequencing of sgTP53-E4.1 and sgTP53-E4.2 clones
6-FAM-/HEX-MK024	[6-FAM]-/[HEX]-TGGTCCTCTGACTGCTCTT	Forward	For fluorescent PCR of sgTP53-E4.1 and sgTP53-E4.2 clones
MK025	GGTGAAGAGGAATCCCAAAGT	Reverse	For SURVEYOR assay and fluorescent PCR of sgTP53-E4.1 and sgTP53-E4.2 clones
MK026	TCTTTCTACACCCACAACTGTAA	Forward	For SURVEYOR assay and sequencing of sgATRX-E2 clones
6-FAM-/HEX-MK026	[6-FAM]-/[HEX]-TCTTTCTACACCCACAACTGTAA	Forward	For fluorescence PCR of sgATRX-E2 clones
MK027	GAAAAAAGACTAGAAGGTATAGCAC	Reverse	For SURVEYOR assay and fluorescent PCR of sgATRX-E2 clones
MK028	TGCCACAGCAACCATGTAA	Forward	For SURVEYOR assay and sequencing of sgATRX-E4 clones
6-FAM-/HEX-MK028	[6-FAM]-/[HEX]-TGCCACAGCAACCATGTAA	Forward	For fluorescence PCR of sgATRX-E4 clones
MK029	TAGTGGTTGACATGAGTTCAGAAA	Reverse	For SURVEYOR assay and fluorescent PCR of sgATRX-E4.1 clones
MK032	CTGAATGAGGCCTTGGAACT	Forward	For RT-PCR of TP53 transcript
MK033	GGCCCTTCTGTCTTGAACAT	Reverse	For RT-PCR of TP53 transcript
MK034	GCCAGACTTATTAGATGACCCTAA	Forward	For RT-PCR of ATRX transcript
MK035	GTTCATGGTATCCTACAATGTGTTC	Reverse	For RT-PCR of ATRX transcript
SL0019	TCACGTCATCCAGCAGAGAATGGA	Forward	For RT-PCR of β-actin transcript (control)
SL0020	CACACGGCAGGCATACTCATCTTT	Reverse	For RT-PCR of β-actin transcript (control)
TD494	GGAGGATTCCAGCGACTC	Forward	For SURVEYOR assay of sgMIR615-T1 to -T4 clones
TD597	[6-FAM]- GGAGGATTCCAGCGACTC	Forward	For fluorescent PCR of sgMIR615-T1 to -T4 clones
TD598	[HEX]- GGAGGATTCCAGCGACTC	Forward	For fluorescent PCR of sgMIR615-T1 to -T4 clones
TD495	GAGAGCCGCAAGACAGG	Reverse	For SURVEYOR assay and fluorescent PCR of sgMIR615-T1 to -T4 clones
001960	(NA)	Both	For RT-PCR of MIR615-3p transcript
002353	(NA)	Both	For RT-PCR of MIR615-5p transcript
001093	(NA)	Both	For RT-PCR of RN46B transcript (control)

Supplementary Table 3. Spacer sequence of sgRNAs

sgRNA	Spacer sequence
sgATRX-E2	TTTTGATTCATTGCAAGTCG
sgATRX-E4	TCGTGACGATCCTGAAGACT
sgTP53-E4.1	GGTGCAGGGGCCGCCGGTGT
sgTP53-E4.2	GGCAGCTACGGTTTCCGTCT
sgMIR615-3p-T1	CTTATTGTTCGGTCCGAGCC
sgMIR615-3p-T2	TTATTGTTCGGTCCGAGCCT
sgMIR615-3p-T3	ACCCTCGAGATCCGAGCACC
sgMIR615-3p-T4	CACCCTCGAGATCCGAGCAC
